# An Update on Hydrogen Sulfide and Nitric Oxide Interactions in the Cardiovascular System

**DOI:** 10.1155/2018/4579140

**Published:** 2018-09-09

**Authors:** Dan Wu, Qingxun Hu, Deqiu Zhu

**Affiliations:** ^1^Department of Pharmacy, Tongji Hospital, Tongji University School of Medicine, Shanghai, China; ^2^Mitochondria and Metabolism Center, Department of Anesthesiology and Pain Medicine, University of Washington, Seattle, USA

## Abstract

Hydrogen sulfide (H_2_S) and nitric oxide (NO) are now recognized as important regulators in the cardiovascular system, although they were historically considered as toxic gases. As gaseous transmitters, H_2_S and NO share a wide range of physical properties and physiological functions: they penetrate into the membrane freely; they are endogenously produced by special enzymes, they stimulate endothelial cell angiogenesis, they regulate vascular tone, they protect against heart injury, and they regulate target protein activity via posttranslational modification. Growing evidence has determined that these two gases are not independent regulators but have substantial overlapping pathophysiological functions and signaling transduction pathways. H_2_S and NO not only affect each other's biosynthesis but also produce novel species through chemical interaction. They play a regulatory role in the cardiovascular system involving similar signaling mechanisms or molecular targets. However, the natural precise mechanism of the interactions between H_2_S and NO remains unclear. In this review, we discuss the current understanding of individual and interactive regulatory functions of H_2_S and NO in biosynthesis, angiogenesis, vascular one, cardioprotection, and posttranslational modification, indicating the importance of their cross-talk in the cardiovascular system.

## 1. Introduction

Hydrogen sulfide (H_2_S) and nitric oxide (NO) are considered as toxic gases and environmental pollutants for many years. However, recent studies investigate that they play a key role in physiological activities in many organ systems. NO, as the first gaseous transmitter, can regulate vascular tone, heart function, endothelial cell angiogenesis, and so on [1, 2]. H_2_S is identified as the third gaseous transmitter due to its biological functions, alongside carbon monoxide (CO), the second transmitter [3].

There are many similar biological characteristics for H_2_S and NO. For example, they are produced by specific enzymes, they penetrate into the membrane freely, and they are sensitive to reactive oxygen species (ROS). Except these features, both molecules regulate many physiological functions through similar signal pathways in the cardiovascular system [4, 5]. Although the interactions between NO and H_2_S are previously considered independently, there is growing evidence of cross-talk between these two gaseous transmitters. In 2009, first experimental evidences reported that there was a cross-talk between NO and H_2_S [6]. Since then, many studies have shown that the biological regulations are dependent on not only NO but also H_2_S. These two molecules can change each other's activities and the interactions alter related proteins' functions [5–7]. The therapeutic potential of NO and H_2_S is very immense and explored through preclinical and clinical studies [8].

Due to the physiological importance of NO and H_2_S, this review discusses the protective effects of NO and H_2_S and the signaling mechanisms under their interactions in the cardiovascular system.

## 2. Physical Properties, Biosynthesis, and Reactivity of H_2_S

### 2.1. Physical Properties of H_2_S

H_2_S is a strong reduced colorless gas with an odor of rotten eggs. H_2_S is easily oxidized to yield some sulfur-containing substances. In aqueous solution, it is hydrolyzed to hydrogen sulfide ions (HS^−^) and sulfide ions (S^2−^), which are in dynamic equilibrium in the following sequential reactions:

(1) $$\begin{array}{c} H_{2}S⇌{HS}^{-}+H^{+}⇌S^{2-}+2H^{+}. \end{array}$$

More than one third of H_2_S is undissociated and the others existed as HS^−^ and S^2−^. The application of H_2_S is most studied in bacteria [9]. Since the discovery of H_2_S generation from mammalian cells, many researchers focus on the biological functions of H_2_S in this emerging field. It is important to investigate the levels of H_2_S in blood and tissue for its physiological functions. There are several analytical methods to detect H_2_S concentration, such as colorimetry [10], fluorescent probes [11], liquid chromatography-mass spectrometry [12], spectrophotometric analysis [13], silver sulfide or polarographic sensor [14, 15], and headspace gas determination [16]. Different analysis technologies got the different H_2_S concentrations. It has been reported that the level of H_2_S in Wistar rats blood is ~10 *μ*mol/L, which was detected by colorimetry method [17], while ~46 *μ*mol/L H_2_S is in Sprague Dawley rat plasma, which was measured by colorimetry method [3]. The plasma level of H_2_S in human is 10–100 *μ*mol/L, which was identified by ion chromatography method [18]. The enzymatic capacity method was used to determine that the physiological plasma level of H_2_S in the brain is 50–160 *μ*mol/L [19]. However, other researchers got different results. Furne et al. used enzymatic capacity method and reported that the H_2_S concentrations in the brain and liver were ~15 nmol/L [20]. Ishigami et al. also showed that the H_2_S concentration was at a low level in the brain, which was detected by gas chromatography method [15]. A new liquid chromatography-mass spectrometry method was developed by Tan et al., and they found that there was ~0.4 *μ*mol/mg protein H_2_S in rat cardiac ventricular myocytes and there was 1.5 *μ*mol/mg protein H_2_S in mice heart [12]. The reason of this inconsistency is that not only H_2_S is easily oxidized but also the disadvantages of analysis technologies are often in question, such as complex preparation processes, low sensitivity and specificity, and time-consuming procedures. Striking different H_2_S concentrations may cause uncertainty for the exact mechanistic role of H_2_S in physiological and pathological processes. Therefore, it is very essential to develop a new method to detect H_2_S concentration in cells, blood, and tissue.

### 2.2. Biosynthesis and Reactivity of H_2_S

**Figure 1:**
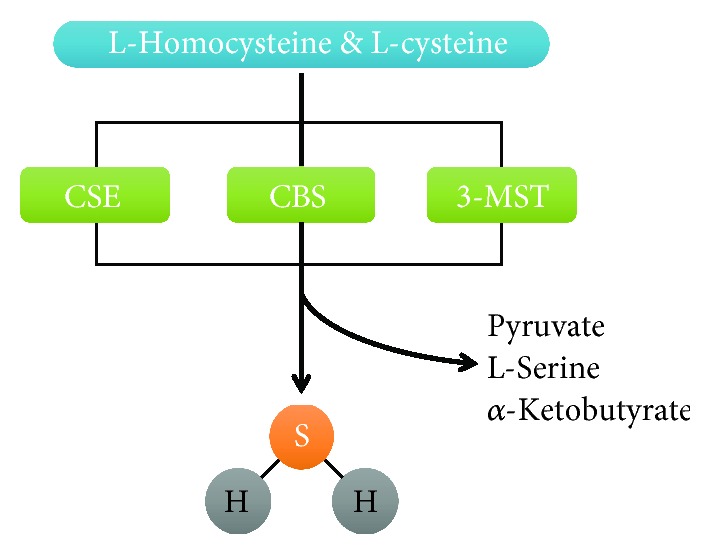
Biosynthesis of H_2_S. L-Homocysteine and L-cysteine are oxidized by three different enzymes, CSE, CBS, and 3-MST, to generate H_2_S.

Endogenous H_2_S is generated in mammalian tissues through enzymatic and nonenzymatic pathways. Two pyridoxal-5′-phosphate- (PLP-) dependent enzymes, cystathionine *γ*-lyase (CSE) and cystathionine *β*-synthase (CBS), use L-cysteine or homocysteine as substrates to synthesize H_2_S [21, 22]. CSE and CBS are expressed in different specific tissues and both of them are needed to produce H_2_S. The expression of CBS is mainly expressed in neurons and astrocytes of the central nervous system (CNS) [19], while CSE is most located in the kidney and liver [23], the cardiovascular system (CVS), especially in cardiomyocytes [24], vascular smooth muscle cells [25], and endothelial cells [26, 27]. In addition, H_2_S is synthesized by 3-mercaptopyruvate sulfurtransferase (3-MST) with cysteine aminotransferase (CAT), which is a PLP-independent pathway for H_2_S formation [28]. The expression of 3-MST is found in the liver, heart, kidney, and brain [29]. CSE and CBS only express in cytosol, while 3-MST expresses not only in cytosol but also in mitochondria. The production of H_2_S from CBS is primary responsible in regulating the nervous system. The H_2_S generation from CSE protects against injuries in the cardiovascular system. CBS catalyzes homocysteine and L-cysteine to generate cystathionine and H_2_S [30]. L-cysteine is catalyzed by CSE into thiocysteine and pyruvate, and then thiocysteine is lysed to produce cysteine and H_2_S [31]. α-Ketoglutarate acid and L-cysteine can be catalyzed by CAT to synthesize 3-mercaptopyruvate. 3-Mercaptopyruvate is desulfurated by 3-MST to generate thiosulfate and then thiosulfate is reduced to produce H_2_S [32] (Figure 1). Some specific inhibitors are available to attenuate the activity of CSE and CBS to reduce the generation of H_2_S, such as D,L-propargylglycine (PAG), *β*-cyano-L-alanine (BCA), aminooxyacetate (AOAA), and hydroxylamine (HA) [33, 34]. PAG can only inhibit the activity of CSE, whereas BCA and AOAA can reduce both activities of CSE and CBS. When at a low concentration, HA is not only an inhibitor of CSE but also an attenuator of the activity of CBS [35–37].

#### 2.2.1. Role of H_2_S in Angiogenesis

**Figure 2:**
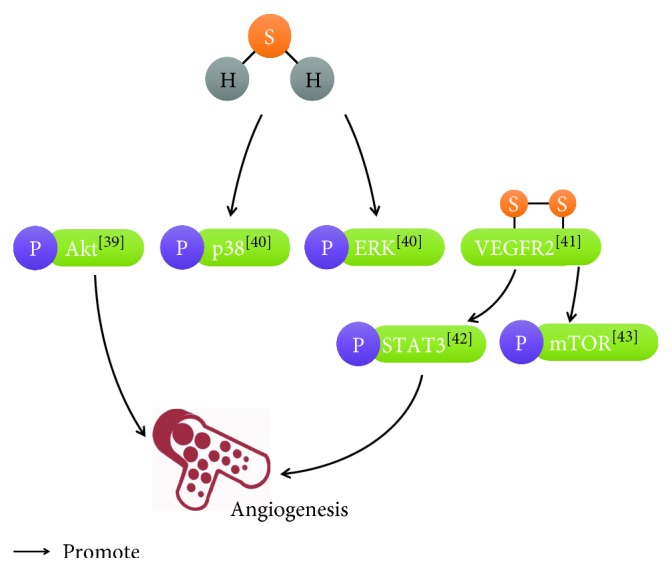
Regulatory role of H2S in endothelial cell angiogenesis. [39]: Cai et al.; [40]: Papapetropoulos et al.; [41]: Tao et al.; [42]: Kan et al.; [43]: Zhou et al.

Ischemic heart disease (IHD) is the main cause of death in the world. There are some weak points in traditional therapeutic methods [38]. H_2_S can stimulate endothelial cell angiogenesis, which is a new potential therapeutic application for IHD. Cai et al. reported that H_2_S could dose-dependently increase the cell number, migration, and tube formation through the Akt pathway [39]. In line with this result, the microvessel formation was obviously inhibited in CSE knockout mice and H_2_S enhanced angiogenesis through the mitogen-activated protein kinase (MAPK) pathway [40]. As a molecular switch, H_2_S specifically broke cys1045-cys1024 disulfide bond in vascular endothelial growth factor receptor 2 (VEGFR2) and stimulated its conformation for angiogenesis [41]. Kan et al. also identified that H_2_S enhanced the activity of signal transducer and activator of transcription 3 (STAT3) through the VEGFR2 pathway [42]. H_2_S increased the mammalian target of rapamycin (mTOR) phosphorylation through VEGFR2 and then stimulated endothelial cell proliferation [43]. However, because of the proangiogenic effect of H_2_S, high concentrations of H_2_S in atherosclerotic plaques will be a potential risk for plaque vulnerability [44] (Figure 2).

#### 2.2.2. Protein S-Sulfhydration by H_2_S

S-sulfhydration is one main posttranslational modification of proteins. Mustafa et al. found that glyceraldehyde-3-phosphate dehydrogenase (GAPDH) was S-sulfhydrated at cys150 by H_2_S to increase catalytic activity [45]. H_2_S S-sulfhydrated Keap1 at cys151 and regulated Nrf2 activation to protect against cellular aging induced by oxidative stress [46]. H_2_S production from CSE S-sulfhydrated the p65 subunit of NF-*κ*B at cys38 to mediate its antiapoptotic actions [47]. Cheung and Lau reported that S-sulfhydrated proteins were identified by proteomic approach in mice aorta, which would be a major step towards understanding the mechanistic role of H_2_S in atherosclerosis. H_2_S also induced S-sulfhydration of glutathione peroxidase 1 and further reduced lipid peroxidation and increased antioxidant defense in the aorta by prompting glutathione synthesis [48]. Endogenous H_2_S, produced by CSE, directly S-sulfhydrated Sirt1 that enhanced Sirt1 binding to zinc ion and then promoted its deacetylation activity and reduced atherosclerotic plaque formation [49]. ATP synthase, the mitochondrial inner membrane protein, regulated mitochondrial bioenergetics, and the *α* subunit (ATP5A1) of ATP synthase was S-sulfhydrated by H_2_S at cys244 and cys294 [50]. H_2_S regulates Krüppel-like factor 5 (KLF5) transcription activity via specificity S-sulfhydration at cys664 to prevent myocardial hypertrophy [51]. S-sulfhydration of specificity protein 1 (Sp1) by H_2_S at cys68 and cys755 plays an important role in maintaining vascular health and function [52]. H_2_S attenuated DNA damage in human endothelial cells and fibroblasts by S-sulfhydrating mitogen-activated protein kinase kinase 1 (MEK1) at cys341, which led to poly[ADP-ribose] polymerase 1 (PARP-1) activation [53]. H_2_S S-sulfhydrated p66Shc at cys59 residue and prevented H_2_O_2_-induced phosphorylation of p66Shc and then inhibited mitochondrial ROS production [54]. Protein tyrosine phosphatases (PTPs) regulate many signal transduction pathways. PTP1B, the one member of PTPs, was reversibly inactivated by H_2_S via S-sulfhydration at cys215 residue [55]. Moreover, H_2_S could S-sulfhydrate and inhibit protein phosphatase 2A (PP2A) to activate 5′ adenosine monophosphate-activated protein kinase (AMPK) in the heart, which resulted to the decrease of mitochondrial biogenesis [56] (Table 1).

#### 2.2.3. Methods Used to Detect Protein S-Sulfhydration

Based on the critical role of S-sulfhydration, various methods are developed to detect the new posttranslational modification of proteins. Biotin-switch assay was firstly used to detect protein nitrosylation, and then this method was modified to determine levels of protein S-sulfhydration. In this assay, S-methyl methanethiosulfonate (MMTS) was used to block thiol group, and N-[6-(biotinamido)hexyl]-30-(20-pyridyldithio) propionamide reacted with the persulfide group. The biotinylated proteins were pulled down by streptavidin agarose beads and detected by Western blot. Using this method, Mustafa et al. reported that about 10–25% proteins were S-sulfhydrated under physiological condition in the liver [45]. However, Pan and Carroll found that MMTS could not block all free thiol groups and caused a false positive [57]. Protein S-sulfhydration was also identified by mass spectrometry (MS) assay. Briefly, target protein was immunoprecipitated and tryptic digested and then injected into the spectrometer [45]. Longen et al. improved MS assay and named it as qPerS-SID (quantitative Persulfide Site Identification). Firstly, trichloroacetic acid was used to dissolve cell completely and ensure cysteine modification stability. Secondly, both thiols and persulfides were labeled by the thiol-reactive reagent, Indoacetyl-PEG2-Biotin, and enriched by streptavidin agarose beads. And then samples were reduced by tris(2-carboxyethyl)phosphine (TCEP) to elute presulfides from the beads selectively before subjected to MS analysis [58].

It was reported that tag-switch assay could selectively detect protein S-sulfhydrated residues. Firstly, methylsulfonyl benzothiazole, −SH block agent, reacted with both –SH and –S-SH to form –S-BT and –S-S-BT. Secondly, –S-S-BT could respond to a biotin-linked cyanoacetate (CN-biotin) to form stable thioether linkages. In the contrast, −S-BT could not be sensitive to CN-biotin. Biotinylated protein could be pulled down by streptavidin agarose beads and then detected by Western blot or MS [59].

Dóka et al. developed the protein persulfide detection protocol (ProPerDP), which could detect S-sulfhydrated protein easily and reliably. In this protocol, both –SH and –S-SH were alkylated by the biotin-labeled alkylating agent, EZ-Link Iodoacetyl-PEG2-Biotin. And then biotinylated proteins were pulled down by streptavidin magnetic beads. Dithiothreitol or TCEP was used to cleave the persulfidated proteins off the beads. Either Western blot or MS was used to detect protein S-sulfhydration, which was dependent on the composition and concentration of samples [60].

#### 2.2.4. Role of H_2_S in Maintenance of Vascular Tone

Studies have shown that H_2_S is a vasorelaxant mediator. The plasma level of H_2_S in hypertension rats was lower than that in normal rats. After injection of H_2_S, the blood pressure was obviously reduced [61]. H_2_S could cause a concentration-dependent relaxation from preconstricted aortic rings. The cyclic guanosine monophosphate (cGMP) level was increased by H_2_S or overexpression of CSE in vasorelaxant process [62]. H_2_S also directly opened the ATP-sensitive K^+^ channel (K_ATP_ channel) for physiological relaxation [25]. H_2_S induced intracellular acidification via activation of Cl^−^/HCO_3_^−^ exchanger, which is partially responsible for H_2_S-mediated vasorelaxation [63]. Metabolic inhibition is also required for the vasorelaxant effects of H_2_S [64]. Nox4 is a positive transcriptional regulator of CSE in endothelial cells and propose that it may in turn contribute to the regulation of vascular tone via the modulation of H_2_S production [65]. H_2_S at low concentrations opened potassuim channels after smooth muscle calcium activated. It also may involve another mechanism, for example, mitochondrial complex I and III led to uncoupling of force, and promoted vasodilation [66].

#### 2.2.5. Role of H_2_S in Heart Protection

**Figure 3:**
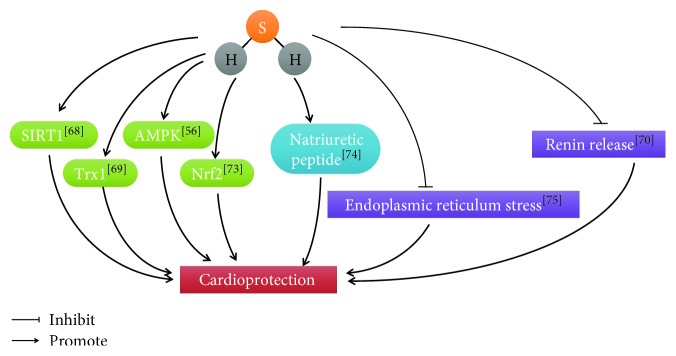
Cardioprotective role of H2S. [68]: Wu et al.; [69]: Nicholson et al.; [70]: Liu et al.; [56]: Shimizu et al.; [73]: Shimizu et al.; [74]: Lilyanna et al.; [75]: Barr et al.

What is more, H_2_S plays an important role to protect against heart failure. The growing evidence indicated that H_2_S levels were decreased in the heart failure of mice [67]. ROS accumulation is a major factor to lead to heart failure. Wu et al. reported that Sirt1 was regulated by H_2_S to reduce ROS for cardiovascular protection [68]. H_2_S also can increase Trx1 to protect against ischemic-induced heart failure [69]. Renin release was inhibited by H_2_S to prevent heart failure [70]. H_2_S may stimulate angiogenesis to regulate cardiac remodeling [71]. H_2_S is a critical regulator of cardiac mitochondrial content and it can promote mitochondrial biogenesis through an AMPK peroxisome proliferator-activated receptor gamma coactivator 1-alpha (PGC1*α*) signaling pathway [56]. H_2_S reduced recruitment of CD11b+Gr-1 cells in mice and has helpful effects on cardiac remolding after myocardial infarction (MI) [72]. H_2_S increased proteasomal activity and function n an Nrf2-dependent manner during the development of heart failure [73]. GYY4137, H_2_S donor, prevented cardiac dysfunction and adverse remodeling through promoting early postischemic endogenous natriuretic peptide activity [74]. H_2_S suppressed endoplasmic reticulum stress stemming from high-fat diet-induced cardiac dysfunction [75] (Figure 3).

## 3. Physical Properties, Biosynthesis, and Reactivity of NO

### 3.1. Physical Properties of NO

Due to the importance of NO in physiological regulation, it was selected as the gaseous transmitter molecule of the year 1992 and the Nobel Prize in Physiology or Medicine was awarded in 1998. NO is a simple molecule with oxygen and nitrogen, which is uncharged. Based on this characteristic, NO penetrates into the membrane freely, which is independent on membrane receptors. NO is also a radical molecule with an unpaired electron. Therefore, NO has a short half-life from 2–30 seconds and it is very reactive to deliver the signal [76].

Although it is difficult to exactly detect NO due to its lability, there are several methods developed to overcome this defect. NO is electrochemical reactive, and electrochemical detection is developed to measure its concentration from cultured cells and rat hearts [77, 78]. Oxyhemoglobin (HbO_2_) can react with NO to yield methemoglobin (metHb) and nitrate (NO_3_^−^), and metHb is measured by spectrophotometric analysis [79]. Nitrogen dioxide (NO_2_) is generated from the reaction of NO with ozone (O_3_), and the light from NO_2_ can be detected, because the excited state of NO_2_ returns to the ground state. NO concentration can also be detected by chemiluminescent methods [80]. Based on the green fluorescent protein, a biosensor is used to measure the level of NO in cells [81].

### 3.2. Biosynthesis and Reactivity of NO

**Figure 4:**
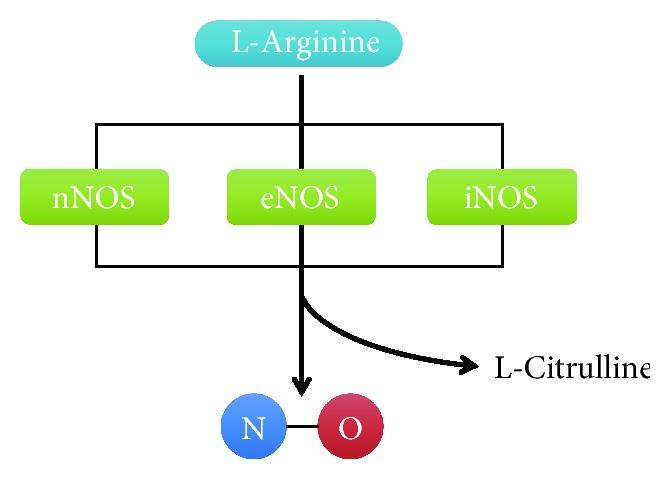
Biosynthesis of NO. L-Arginine is catalyzed by three different enzymes, nNOS, eNOS, and iNOS, to produce NO.

NO is enzymatically synthesized by the NO synthase (NOS) family of proteins [82]. There are three distinct isoforms of NOS proteins, neuronal NOS (nNOS) (encoded by NOS1), endothelial inducible NOS (iNOS) (encoded by NOS2), and NOS (eNOS) (encoded by NOS3). These three NOSs are oxidoreductase homodimer enzymes including two domains, an amino-terminal oxygenase domain and a reductase domain. The amino-terminal domain contains three binding sites for a ferric haem cluster, cofactor tetrahydrobiopterin (BH4) and substrate L-arginine. The reductase domain includes three binding sites for flavin mononucleotide (FMN), flavin adenine dinucleotide (FAD), and the electron donor nicotinamide adenine dinucleotide phosphate (NADPH). These two domains are linked by a sequence that binds calcium-complexed calmodulin [83]. Upon NOS activation, FAD and FMS transfer electrons from NADPH to heme. Reductase domains of monomers bind calmodulin and then boost the transfer of electrons. The electrons promote binding of O_2_ to the ferrous through reduced haem iron. L-Arginine binds the ferrous form to generate L-citrulline and NO [84–86] (Figure 4).

nNOS is the first NOS to be cloned and is mainly expressed in the sarcoplasmic reticulum (SR) of cardiac myocytes [87], in autonomic cardiac neurons and ganglia [88], and within vascular smooth muscle cells (VSMCs) [89, 90]. eNOS is highly expressed not only in the endothelial cells but also in cardiac myocytes [91, 92] and platelets [93]. iNOS can be found in a lot of cell types, such as leukocytes, endothelial cells, VSMCs, cardiac myocytes, nerve cells, and fibroblasts [94, 95]. Increased intracellular Ca^2+^ levels could promote eNOS and nNOS to produce NO. Unlike eNOS and nNOS, NO generation from iNOS is calcium independent. Inflammatory stimuli easily induce the expression of iNOS, such as cytokine (TNF-*α* or IFN-*γ*), bacterial proteins/peptides, or lipopolysaccharide (LPS). The dimeric enzyme catalytic activity of iNOS is much higher than those of nNOS and eNOS, when NOSs are assembled. iNOS maintains larger quantity of NO until exhaustion of substrate and cofactors or enzyme degradation; therefore, elevated iNOS expression is mostly associated with pathological stress [2, 84, 96, 97]. In addition, NO^3−^ and nitrite (NO^2−^), which exist in diet, generate NO through the nonenzymatical pathway. Therefore, NO can be supplied through daily diet [98]. S-Nitroso-L-glutathione (GSNO) is another form of NO storage. GSNO releases NO through catalyzing enzymes, such as GSH peroxidase and thioredoxin reductase [99, 100].

#### 3.2.1. Role of NO in Angiogenesis

**Figure 5:**
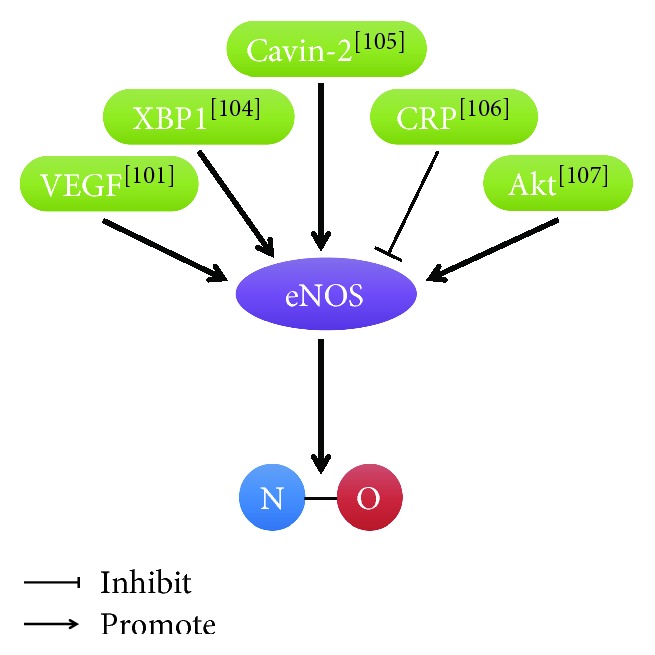
Regulatory role of NO in endothelial cell angiogenesis. [101]: Fukumura et al.; [104]: Yang et al.; [105]: Boopathy et al.; [106]: Verma et al.; [107]: Dimmeler et al.

NO has an important role for angiogenesis. Vascular endothelial growth factor (VEGF) upregulated eNOS expression and increased NO release to stimulate angiogenesis [101]. VEGF-induced cell proliferation was attenuated by NG-nitro-L-arginine methyl ester (L-NAME) [102, 103]. X-Box binding protein 1 (XBP1) stimulated endothelial cell migration via regulating eNOS expression [104]. Cavin-2 promoted the generation of NO in endothelial cells by controlling activity of eNOS and then stimulated endothelial cell angiogenesis [105]. C-reactive protein (CRP) quenched the production of NO through posttranscriptional effect on eNOS mRNA stability and then inhibited angiogenesis [106]. Phosphorylation of eNOS and NO production were mediated by Akt and then regulated angiogenesis [107] (Figure 5).

#### 3.2.2. Proteins S-Nitrosylation by NO

S-nitrosylation is one main way of NO to mediate protein activity. NO can S-nitrosylate G protein-coupled receptor kinases (GRKs) to suppress their activity and block phosphorylation [108]. p65 subunit of NF-*κ*B is S-nitrosylated at cys38 to protect against inflammation [109]. S-nitrosylated arginase1 contributed to endothelial dysfunction in the aging cardiovascular system [110]. NO S-nitrosylated N-ethylmaleimide-sensitive factor (NSF) and then inhibited exocytosis of Weibel-Palade bodies [111]. Using transgenic mice to titrate the levels of S-nitrosylation protein, Irie et al. uncovered major roles for protein S-nitrosylation generally and for phospholamban (PLN) and cardiac troponin C (cTnC) S-nitrosylation in particular, in *β*-AR-dependent regulation of Ca^2+^ homeostasis [112]. Dynamic S-nitrosylation/denitrosylation of *β*-arrestin 2 regulated stimulus-induced GPCR trafficking [113]. nNOS deficiency impaired ryanodine receptor (RyR) S-nitrosylation and led to altered Ca^2+^ homeostasis [114]. S-nitrosylation of native transient receptor potential channel 5 (TrpC5) at cys553 and nearby cys558 upon G protein-coupled ATP receptor stimulation elicited entry of Ca^2+^ into endothelial cells [115]. S-nitrosylation at the cys215 residue of PTP1B protected against H_2_O_2_-induced irreversible oxidation [116]. S-nitrosylation of GAPDH triggered binding to Siah1, an E3 ubiquitin ligase, nuclear translocation, and cell apoptosis [117] (Table 2).

#### 3.2.3. Role of NO in Maintenance of Vascular Tone

The primary function of NO is identified as endothelial-derived relaxation factor (EDRF) [118, 119]. Endothelial cells can produce small quantities of NO to stimulate vascular smooth muscle relaxation. Due to short half-life, vasoconstriction does not happen unless persistent NO has been generated. NOS inhibitors are used to investigate the physiological roles of NO in biological systems, such as L-N-mono-methyl-arginine (L-NMMA) and L-NAME. When inhibitors are added, NO is attenuated and blood pressure increases. Increase of blood flow enhances NO production and causes vessel relaxation. So, NO also is an endogenous mediator of blood flow [2, 120]. NO regulated adrenomedullin-induced vasorelaxation via the cGMP pathway [121]. NO stimulated Ca^2+^-dependent K^+^ channel activity and opened Ca^2+^-dependent K^+^ channels to induce relaxation of vascular smooth muscle cells [122]. NO deficit increased vascular tone in the contribution of Cav3.1 and Cav3.2 T-type calcium channels through regulating the bioavailability of ROS produced by NADPH oxidase [123].

#### 3.2.4. Role of NO in Heart Protection

**Figure 6:**
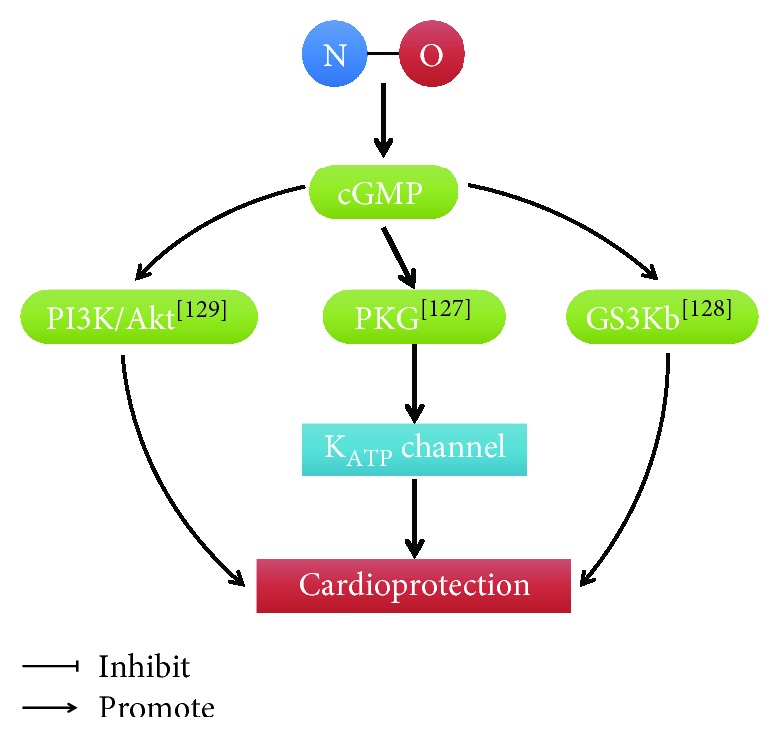
Cardioprotective role of NO. [127]: Xu et al.; [128]: Bhushan et al.; [129]: Couto et al.

Congestive heart failure results in cardiovascular dysfunction and diminishes vascular NO production. Targeted overexpression of the eNOS gene within the vascular endothelium in mice attenuated both cardiac and pulmonary dysfunction and dramatically improves survival during severe congestive heart failure [124]. During ischemia/reperfusion, the more serious heart functions were found in eNOS-deficient mice compared with wild-type mice [125]. NO is an important modulator of left ventricular (LV) remodeling after myocardial infarction (MI). Cardiomyocyte-restricted overexpression of eNOS limited LV dysfunction and remodeling after MI [126]. NO stimulated PKG activity and opened the K_ATP_ channel to induce ROS generation in cardiomyocytes [127]. Nitrite increased NO levels and prevented the progression of hypertrophy and heart failure via cGMP/GS3K*β* signaling [128]. The coronary arteries from the heart failure rats exhibited reduced NO bioavailability, whereas the MI rats exhibited increased NO bioavailability because of the increased eNOS/nNOS/PI3K/Akt pathway and a reduction in ROS generation [129] (Figure 6).

## 4. Cross-Talk between H_2_S and NO

In the past several years, attention has been given to gas cross-talk. Growing evidence has shown that these two gasotransmitters interact with each other's biosynthesis and physiological response in many ways. However, there is still no clarity about the nature of the interaction [7, 130]. H_2_S and NO can affect not only the generation of each other through enzymatic expression and activity but also the further downstream signaling pathway [131–133]. The next part will provide current understanding of the interactions and mechanisms between H_2_S and NO in the cardiovascular system.

### 4.1. Biosynthesis of H_2_S and NO Interaction

**Figure 7:**
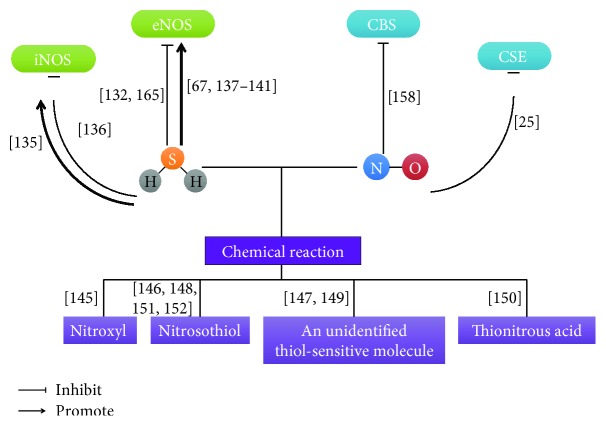
The interactions between H2S and NO on biosynthesis and chemical reaction. [25]: Zhao et al.; [67]: Kondo et al.; [132]: Kubo et al.; [135]: Hua et al.; [136]: Jeong et al.; [137]: Predmore et al.; [138]: Kida et al.; [139]: Altaany et al.; [140]: Sojitra et al.; [141]: King et al.; [145]: Eberhardt et al.; [146]: Whiteman et al.; [147]: Yong et al.; [148]: Ali et al.; [149]: Yong et al.; [150]: Filipovic et al.; [151]: Ondrias et al.; [152]: Cortese-Krott et al.; [158]: Wang et al.; [165]: Geng et al.

The activity of CBS was suppressed by NO through binding to the enzyme. A five-coordinate ferrous nitrosyl species was formed and ligands are lost [134]. H_2_S directly inhibited the activity of recombinant eNOS to cause the increase of aortic contractility. However, the natural mechanism is not clear [132]. In the model of myocarditis, there were high iNOS mRNA and protein expression. H_2_S therapy inhibited iNOS overexpression to limit inflammatory cell infiltration, suppress cardiac edema, and attenuate myocardial lesions [135]. These two gasotransmitters inhibit each other's production; however, it has been shown that NO and H_2_S promote their respective synthesis. NO obviously increased CSE expression and H_2_S generation from vascular tissues. The dose-dependent relaxation curve of H_2_S was shifted to the right by L-NAME [25]. Likely, H_2_S enhanced iNOS expression and NO production through the IL-1*β*-induced NF-*κ*B signaling pathway [136]. In endothelial cells, H_2_S increased NO generation twofold from eNOS and eNOS was activated at Ser 1177 through the Akt pathway [137]. It was also found that H_2_S obviously increased calcium concentrations and activated eNOS at phosphoserine residue 1179. A calcium chelator abolished H_2_S-induced NO synthesis in endothelial cells. So, eNOS activation and NO generation were regulated by H_2_S through calcium release [138]. Altaany et al. reported that although H_2_S stimulated eNOS activation, the expression of eNOS is slightly affected by H_2_S in endothelial cells. NO production was inhibited by CSE knockdown, whereas CSE overexpression enhanced NO generation [139]. H_2_S concentrations were decreased in heart failure. H_2_S therapy regulated eNOS and increased NO production to protect against heart failure [67]. In isoproterenol-induced myocardial injury, H_2_S played a cardioprotective role but this effect was abrogated when NOS was inhibited [140]. In CSE KO mice, H_2_S reduction caused eNOS dysfunction, limitation of NO production, and elevated oxidative stress. When using exogenous H_2_S therapy, eNOS was activated and NO levels were increased and oxidative stress was obviously inhibited. However, H_2_S did not diminish oxidative stress injury in eNOS phosphomutant mice. Therefore, H_2_S-mediated cytoprotection was closely correlated to eNOS activation and NO generation [141] (Figure 7).

### 4.2. Posttranslational Modifications of H_2_S and NO Interaction

**Figure 8:**
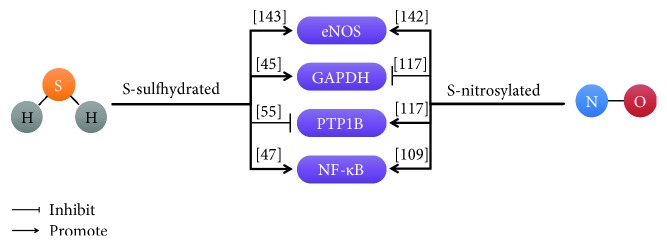
The interactions between H2S and NO on the same protein targets. [45]: Mustafa et al.; [47]: Sen et al.; [55]: Krishnan et al.; [109]: Kelleher et al.; [117]: Hara et al.; [142]: Altaany et al.; [143]: Bir et al.

S-sulfhydration and S-nitrosylation are important mechanisms for H_2_S and NO, respectively, to modify target protein. H_2_S could enhance eNOS activity by S-sulfhydration. There are both monomeric eNOS and dimeric eNOS in cells, but NO is produced only by eNOS dimers. H_2_S promoted eNOS dimer formation to increase NO generation. Cys443 in eNOS was not only a S-sulfhydration site but also a S-nitrosylation site. S-sulfhydration of eNOS was not affected by NO, whereas H_2_S inhibited the S-nitrosylation of eNOS [142]. In chronic tissue ischemia, H_2_S increased NO generation through eNOS activity and nitrite reduction mechanism [143]. Cys215 in PTP1B was also both S-nitrosylated and s-sulfhydrated. S-nitrosylation of PTP1B could increase its activity, but s-sulfhydration of PTP1B could prevent its activity [55, 116]. Similarly, GAPDH was also modified by both S-nitrosylation and s-sulfhydration at cys150. NO inhibited the activity of GAPDH, while H_2_S increased the activity of GAPDH [45, 117]. Intriguingly, the p65 subunit of NF-*κ*B at cys38 was both S-nitrosylated and s-sulfhydrated, and either NO or H_2_S could increase NF-*κ*B activity [47, 109]. NaHS treatment on reperfusion increases S-nitrosylation to a level comparable to that with S-nitroso-N-acetylpenicillamine (SNAP) treatment. In addition, there was an additive increase in S-nitrosylation but not S-sulfhydration when SNAP and NaHS were added together at reperfusion. Thus, part of the benefit of sodium hydrosulfide (NaHS) is an increase in S-nitrosylation and the magnitude of the protective effect is related to the magnitude of the increase in S-nitrosylation [144]. Taken together, S-sulfhydration and S-nitrosylation at the same cys residues of target protein dynamics balance and compete with each other to maintain normal function of the protein (Figure 8).

### 4.3. Biochemistry of H_2_S and NO Interaction

Although above evidences indicate that these two gases affect each other's synthesis, other researchers suggest that H_2_S and NO chemical interact to from a novel molecule. Nitroxyl (HNO) is a sibling of NO that releases calcitonin gene-related peptide (CGRP) to play a cardioprotective role. Eberhardt et al. showed that H_2_S interacted with NO to form HNO. NO and H_2_S converged at transient receptor potential channel A1 (TRPA1). TRPA1 was activated by HNO through the formation of disulfide bonds and CGRP was released to regulate vascular tone [145]. It also has been shown that a novel nitrosothiol was produced by a direct interaction between NO and H_2_S, which was detected by a combination of analysis methods. This result was also identified in the LPS-treated liver. The nitrosothiol did not increase cGMP concentration without Cu^2+^ in RAW264.7 cells [146]. H_2_S and NO play a key role to regulate heart function. The extent of myocyte contraction was reduced by NO, while H_2_S also had small effect to stimulate heart contractility. However, H_2_S + NO did not play an inotropic role in the presence of thiols. This result suggested that H_2_S may interact with NO to generate a new molecule, which was very sensitive to thiols. This new thiol-sensitive molecule has not been identified, but it exerts significant regulatory role in the heart. Further work needs to be done to explain this question and offer new therapeutic application [147]. Ali et al. reported that H_2_S and NO might interact together to produce an unidentified nitrosothiol, which inhibited vasorelaxant potential of NO not only *in vitro* but also *in vivo*. The authors also proposed that, although H_2_S had vasorelaxant activity, the crucial function of H_2_S was to regulate local levels of NO [148]. A thiol-sensitive molecule was formed by the chemical interaction between H_2_S and NO, which had positive inotropic and lusitropic effects [149]. S-Nitrosothiols could interact with H_2_S to produce thionitrous acid (HSNO), which provided NO^+^, NO, and NO^−^ to play a physiological role [150]. Ondrias et al. reported that nitrosothiols reacted with H_2_S to release NO to exert biological functions [151]. When nitrosothiol interacted with sulfide in excess, SSNO^−^ was obtained, which was very stable at physiological pH and produced polysulfides and NO [152] (Figure 7).

### 4.4. Role of the Interaction of H_2_S and NO in Angiogenesis

**Figure 9:**
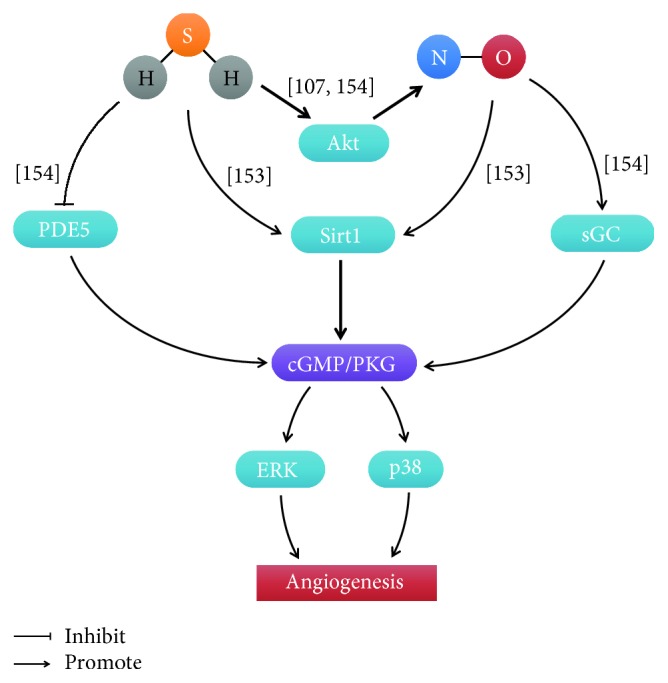
The interactions between H2S and NO in endothelial cell angiogenesis. [107]: Dimmeler et al.; [153]: Hu et al.; [154]: Coletta et al.

Accumulating evidences report that H_2_S and NO play an important role in endothelial cell angiogenesis. However, the mechanisms of interaction between H_2_S and NO are still exclusive. We found that H_2_S and NO converged at the same downstream molecular target, Sirt1. Sirt1 activation increased the VEGF level and cGMP concentration. Evoked by the increase in cGMP levels, cGMP/PKG and downstream molecules, including p38 and ERK, were activated to participate in the regulation of angiogenesis [153]. Coletta et al. also reported that both NO and H_2_S could increase intracellular cGMP. H_2_S decreased cGMP degradation by preventing phosphodiesterase type 5 (PDE5), while NO activated sGC to produce cGMP [154]. As mentioned previously, H_2_S stimulated angiogenesis via Akt phosphorylation. eNOS activity was induced by the increase of Akt phosphorylation [107]. It predicted that H_2_S activated Akt and increases eNOS phosphorylation at its activating site Ser1177 [154] (Figure 9).

### 4.5. Role of the Interaction between H_2_S and NO in Vascular Tone

Several studies have shown that the interactions between H_2_S and NO maintain vascular tone. Hosoki et al. firstly found that H_2_S induced much stronger vascular relaxation in the presence of NO [155]. The vascular denervation did not affect H_2_S-induced vasorelaxation; however, the vasorelaxant effect of H_2_S was inhibited in the absence of endothelium. When the nitric oxide synthase was blocked, H_2_S-induced vasorelaxation was attenuated [25]. We also found that H_2_S and NO generation from ZYZ-803, a novel H_2_S- and NO-conjugated donor, cooperatively regulated vascular tone through the cGMP pathway. Either blocking CSE and/or eNOS activity, or uncontaining endothelium could prevent ZYZ-803-induced vasorelaxation [156]. In line with these results, Coletta et al. reported that H_2_S and NO are mutually dependent to regulate endothelium-dependent vasorelaxation [154]. The data above showed that H_2_S have a vasoregulatory role in a NO-dependent manner. However, the regulatory role of H_2_S and NO in vasorelaxation was differently found by other labs. 1H-oxadiazolo-quinoxalin-1-one (ODQ) and NS-2028 were cGMP inhibitors, which suppressed SNP-induced relaxation. But ODQ and NS-2028 could promote H_2_S-induced vasorelaxation. Moreover, the vasorelaxant potent of SNP was reduced when aortic tissues were pretreated with H_2_S [157]. Similarly, Whiteman et al. [146] and Ali et al. [148] found that the vasorelaxant effect of NO was inhibited by H_2_S. Wang et al. reported that PAG inhibited the relaxant effect of NO [158].

### 4.6. Role of the Interaction of H_2_S and NO in Heart Protection

**Figure 10:**
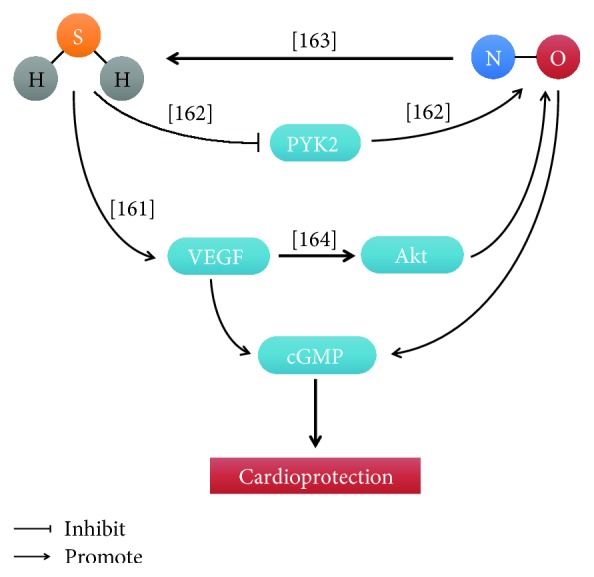
The interactions between H2S and NO in cardioprotection. [161]: Wu et al.; [162]: Bibli et al.; [163]: Donnarumma et al.; [164]: Karwi et al.

Both H_2_S and NO have a cardioprotective role in the heart. H_2_S protected against ischemic injury via increasing NO release and adding L-NAME attenuated the cardioprotective of H_2_S [159]. Sojitra et al. also found that H_2_S alleviated isoproterenol-induced cardiomyopathy through elevating myocardial and serum NO levels and inhibition of NOS activity abrogated the cardioprotective role of H_2_S [140]. H_2_S postconditioning conferred the protective effects against ischemia-reperfusion injury through the activation of eNOS pathways [160]. Similarly, CSE KO mice exhibited reduced levels of NO and reduced NO synthesis via eNOS, which increased oxidative stress and an exacerbated response to myocardial ischemia/reperfusion injury [141]. We also found that H_2_S and NO cooperatively attenuated left ventricular remodeling and dysfunction during the development of heart failure through the VEGF/cGMP pathway [161]. A novel H_2_S donor, SG-1002, prevented the transition from compensated to decompensated heart failure in part via upregulation of eNOS and increased nitric oxide bioavailability [67]. Bibli et al. reported that H_2_S preserved eNOS activity via inhibiting proline-rich tyrosine kinase 2 (PYK2) in H9c2 cells under oxidative stress [162]. Sodium nitrite (NaNO_2_) significantly improves LV function in ischemia-induced chronic heart failure via increasing H_2_S bioavailability, Nrf2 activation, and antioxidant defenses [163]. GYY4137, a slow-releasing H_2_S donor, protected the heart against lethal reperfusion injury through activation of the PI3K/Akt pathway, with partial dependency on NO [164]. However, the other labs reported some conflicting results. Kubo et al. showed that the activity of eNOS was inhibited by H_2_S in rat and mouse aortic rings [132]. In addition, Geng et al. found that both exogenous and endogenous H_2_S reduced NO generation and prevented eNOS activity and transcription [165]. This is possible that differences of H_2_S concentration and experimental model cause the conflicting result and the interaction between H_2_S and NO in heart protection is still needed to study (Figure 10).

## 5. Conclusion

In this review, we have summarized the biological functions of H_2_S and NO and described the interactions between these two gases in the cardiovascular system. As gasotransmitters, there are some similar functions between H_2_S and NO. They do not only have similar biological reactivity but also have similar biological effects. H_2_S and NO interact with each other's synthesizing enzymes and affect their generation. Moreover, H_2_S and NO directly produce a new unidentified compound by chemical interaction. Both of them are endothelial-derived relaxation factors to regulate vascular tone. They also stimulate endothelia cell angiogenesis and protect against heart injury. In addition, H_2_S and NO regulate target protein activity through S-sulfhydration and S-nitrosylation at special cysteine residue to exert biological effects.

It is beyond debate that either H_2_S or NO plays a critical role in regulation of the mammalian cardiovascular system. Although accumulating evidence has suggested that H_2_S and NO interact with each other in the cardiovascular system, the natural precise mechanism of the interactions remains unclear. Both H_2_S and NO work on each other's physiological generation and response in the cardiovascular system; however, these conclusions sometimes appear inconsistent. This may be caused by different gasotransmitter donors and levels, different experimental models and parameters, and so on. Some groups have found that H_2_S and NO chemically produced a novel compound, like HNO. But another group also showed that H_2_S and NO interaction generated other chemical species. This will be needed to develop novel methods or instruments to measure these unknown species. Moreover, H_2_S and NO can modify the same target protein even at the same cysteine residue, but the competitive mechanism is still covered and how the posttranslational modifications affect target protein activity. Although there is plenty of information pointing towards a physiological regulation of H_2_S and NO, much work needs to be done to investigate the cross-talk between H_2_S and NO. A deeper understanding of the interactions will cause the development of novel therapeutic strategies for cardiovascular diseases.

## Figures and Tables

**Table 1:** Selected protein targets of H2S.

Selected protein activity	Activity
GAPDH	Increased [45]
Keap1	Decreased [46]
NF-*κ*B	Increased [47]
Glutathione peroxidase 1	Increased [48]
Sirt1	Increased [49]
ATP5A1	Increased [50]
KLF5	Decreased [51]
Sp1	Increased [52]
MEK1	Increased [53]
p66Shc	Decreased [54]
PTP1B	Decreased [55]
PP2A	Decreased [56]

[45]: Mustafa et al.; [46]: Yang et al.; [47]: Sen et al.; [48]: Cheung et al. and Lau; [49]: Du et al.; [50]: Modis et al.; [51]: Meng et al.; [52]: Saha et al.; [53]: Zhao et al.; [54]: Xie et al.; [55]: Krishnan et al.; [56] Shimizu et al.

**Table 2:** Selected protein targets of NO.

Selected protein activity	Activity
GPCR	Decreased [108]
NF-*κ*B	Increased [109]
Arginase1	Increased [110]
NSF	Increased [111]
PLN	Increased [112]
cTnC	Increased [112]
*β*-Arrestin 2	Decreased [113]
RyR	Increased [114]
TRPC5	Increased [115]
PTP1B	Decreased [116]
GAPDH	Decreased [117]

[108]: Whalen et al.; [109]: Kelleher et al.; [110]: Santhanam et al.; [111]: Matsushita et al.; [112]: Irie et al.; [113]: Ozawa et al.; [114]: Gonzalez et al.; [115]: Yoshida et al.; [116]: Chen et al.; [117]: Hara et al.

## Abbreviations

3-MST: 3-Mercaptopyruvate sulfurtransferase
AMPK: 5′ adenosine monophosphate-activated protein kinase
AOAA: Aminooxyacetate
BCA: *β*-Cyano-L-alanine
BH4: Tetrahydrobiopterin
CAT: Cysteine aminotransferase
CBS: Cystathionine *β*-synthase
cGMP: Cyclic guanosine monophosphate
CGRP: Calcitonin gene-related peptide
CN: Cyanoacetate
CNS: Central nervous system
CO: Carbon monoxide
CRP: C-reactive protein
CSE: Cystathionine *γ*-lyase
cTnC: Cardiac troponin C
CVS: Cardiovascular system
EDRF: Endothelial-derived relaxation factor
eNOS: Endothelial NOS
FAD: Flavin adenine dinucleotide
FMN: Flavin mononucleotide
GAPDH: Glyceraldehyde-3-phosphate dehydrogenase
GRKs: G protein-coupled receptor kinases
GSNO: S-Nitroso-L-glutathione
H_2_S: Hydrogen sulfide
HA: Hydroxylamine
HbO_2_: Oxyhemoglobin
HNO: Nitroxyl
HS^−^: Hydrogen sulfide ions
IHD: Ischemic heart disease
iNOS: Inducible NOS
K_ATP_ channel: ATP-sensitive K^+^ channel
KLF5: Krüppel-like factor 5
L-NAME: L-NG-Nitroarginine methyl ester
L-NMMA: L-N-Mono-methyl-arginine
LPS: Lipopolysaccharide
LV: Left ventricular
MAPK: Mitogen-activated protein kinase
MEK1: Mitogen-activated protein kinase kinase 1
metHb: Methemoglobin
MI: Myocardial infarction
MMTS: S-Methyl methanethiosulfonate
MS: Mass spectrometry
mTOR: Mammalian target of rapamycin
NADPH: Nicotinamide adenine dinucleotide phosphate
NaHS: Sodium hydrosulfide
NaNO_2_: Sodium nitrite
nNOS: Neuronal NOS
NO: Nitric oxide
NO_2_: Nitrogen dioxide
NO^2−^: Nitrite
NO_3_^−^: Nitrate
NOS: NO synthase
NSF: N-Ethylmaleimide-sensitive factor
ODQ: 1H-Oxadiazolo-quinoxalin-1-one
O_3_: Ozone
PAG: D,L-Propargylglycine
PARP-1: Poly[ADP-ribose] polymerase 1
PDE5: Phosphodiesterase type 5
PGC-1*α*: Peroxisome proliferator-activated receptor gamma coactivator 1-alpha
PLN: Phospholamban
PP2A: Protein phosphatase 2A
PTPs: Protein tyrosine phosphatases
PYK2: Proline-rich tyrosine kinase 2
ROS: Reactive oxygen species
RyRs: Ryanodine receptors
S^2−^: Sulfide ions
SNAP: S-Nitroso-N-acetylpenicillamine
Sp1: Specificity protein 1
SR: Sarcoplasmic reticulum
STAT3: Signal transducer and activator of transcription 3
TCEP: Tris(2-carboxyethyl)phosphine
TRPA1: Transient receptor potential channel A1
TrpC5: Transient receptor potential channel 5
VEGF: Vascular endothelial growth factor
VEGFR2: Vascular endothelial growth factor receptor 2
VSMCs: Vascular smooth muscle cells
XBP1: X-Box binding protein 1.
